# Interpretation discrepancies of abdominal imaging by on-call radiology residents: Evaluation of risk factors

**DOI:** 10.1371/journal.pone.0274313

**Published:** 2022-09-09

**Authors:** Su Jeong Yang, Hee Joong Lim, So Hyun Park, Seung Joon Choi, Young Sup Shim

**Affiliations:** Department of Radiology, Gil Medical Center, Gachon University College of Medicine, Namdong-gu, Incheon, Korea; University Magna Graecia of Catanzaro, ITALY

## Abstract

The aim of this study was to determine the rate, important findings, and risk factors related to discrepancies between on-call residents’ and attending radiologists’ interpretations of abdominal examinations. We identified 1132 eligible patients with abdominal radiology findings that were preliminary interpreted by on-call residents between February 2016 and September 2019. The preliminary interpretations were compared with the final interpretations by abdominal attending radiologists, including clinical data. The preliminary interpretations were analyzed by three radiologists in consensus, who categorized the reports according to organs, important findings (i.e., active bleeding, bowel obstruction, organ ischemia or infarction, and organ rupture), clinical outcomes, and discrepancies with respect to final interpretations. Multiple logistic regression analysis was used to evaluate the risk factors for important discrepant findings. Of 1132 patients, the bowel (*n* = 567, 50.1%) was the most common organ interpreted by on-call residents, followed by gallbladder/bile duct/pancreas (*n* = 139, 12.3%) and liver (*n* = 116, 10.2%). Of 1132, 359 patients (31.7%) had disease with 379 important findings: active bleeding (*n* = 222), organ rupture (*n* = 77), bowel obstruction (*n* = 52), bowel ischemia (*n* = 24), and organ infarction (*n* = 4). Sixty-four patients (5.6%) showed discrepancies, and 30 (2.6%) showed 32 important discrepant findings comprising 14 active bleeding, 10 bowel obstructions, 6 organ ruptures, and 2 cases of bowel ischemia. Of the 64 discrepant patients, 33 underwent delayed surgery (*n* = 18, 28.1%) or interventional treatment (*n* = 15, 23.4%). In multivariable analysis, bowel obstruction (adjusted odds ratio, 2.52; *p* = 0.049) was an independent risk factor for determining discrepancy between preliminary and final interpretations. The rate of overall and important discrepancies between on-call residents’ and final interpretations was low. However, given that the bowel was the most frequently interpreted organ, bowel obstruction was identified as a risk factor for discrepant interpretations. The identified risk factor and findings may be useful for residents to minimize discrepancies.

## Introduction

In many academic radiology departments, radiology residents often provide after-hour coverage for preliminary independent radiology examinations performed on inpatients and conducted in the emergency department. A common evaluation by on-call radiology residents is conducted on abdominal examinations, which are often challenging, and attending radiologists review these interpretations the next morning.

Many previous studies have reported low rates of discrepancies between the preliminary report from residents and the final report by attending radiologists [1–7]. Nevertheless, diagnostic errors in preliminary radiology reports may cause discrepancies. Errors are divided into 1) perceptual (misses) errors and 2) interpretation (differential diagnosis) errors [8]. Identifying the underlying risk factors or causes of erroneous evaluation may lead to reduce the discrepancy rate. However, to the best of our knowledge, no study has investigated the risk factors for discrepancies between preliminary abdominal radiology reports provided by residents and the final reports verified by attending radiologists. As misinterpretations during overnight duty may result in changes to treatments and additional evaluations [3], it is important to analyze discrepancies between preliminary and final reports in abdomen radiology studies.

We conducted a retrospective review of preliminary reports of abdominal imaging examinations by radiology residents during after-hour coverage. We analyzed the discrepant cases and risk factors for discrepancies between residents’ preliminary reports and attending radiologists’ final reports along with the clinical outcomes. If residents can identify such discrepant cases and risk factors for discrepancies and prepare for similar situations before on-call duty, misinterpretations may be reduced, thus improving diagnostic accuracy of preliminary readings.

We aimed to determine the rate, types, important findings, and risk factors related to discrepancies between residents’ preliminary reports and final interpretations by abdominal attending radiologists including the clinical outcomes.

## Materials and methods

This retrospective study at a tertiary referral center was approved by the Institutional Review Board of Gil medical center (GAIRB2021-378), and the requirement for obtaining written informed patient consent was waived.

### Study population

**Fig 1:**
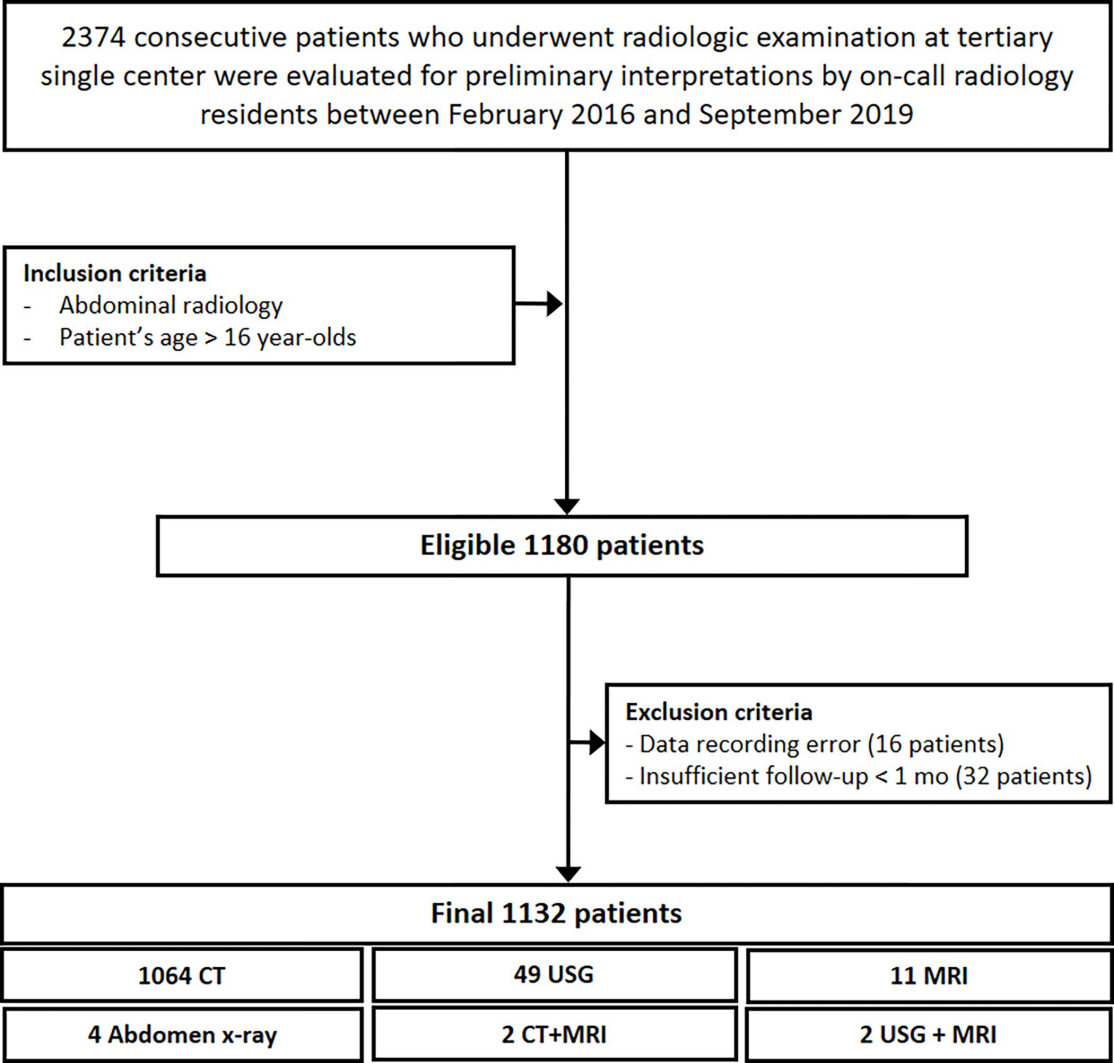
Patient selection flowchart.

We evaluated 2374 consecutive patients for the preliminary radiology interpretations by on-call radiology residents between February 2016 and September 2019. From this overall set, we identified eligible patients that were over 16 years of age and with a consultation which included abdominal imaging. Among 1180 eligible patients, 16 patients had data recording errors and 32 patients had insufficient follow-up time (less than one month) and were excluded. For this study, we reviewed data on 1132 patients with preliminary radiology interpretations made by on-call radiology residents (Fig 1).

### Preliminary report data by resident

In our institution, radiology residents take on-call duty from 5 pm to 8 am on weekdays and 8 am to 8 am overnight on weekends and holidays for emergency department and inpatient examinations. The evaluations are based on the referring clinician’s questions made by phone call regarding simple radiography, ultrasonography (US), CT, and MRI. The questions regarding simple radiography, CT, and MRI are interpretative, and those on US query about the possibility of the resident to perform on-call US and interpret it. The residents in our institution begin taking on-call duty responsibilities between the second half of the first year and the first half of the fourth year of training. The duty consists of mainly second- and third-year job (approximately 85–90%) and remnant job of the second half of the first year and the first half of the fourth year. All residents are educated about abdominal radiology for more than 8 weeks before taking a call. In some cases, when a junior (first-year) resident has difficulty with a case during on-call duty, they can ask a senior resident regarding the case. At the end of each overnight shift, all residents should record a list containing the consulted patients, their information, and preliminary reports for educational purposes in our institution’s database. The database includes the patients’ sex, age, number, image study date, date of duty, name of on-call duty resident, and reason for the consultation.

### Evaluating discrepancies

The complete database was reviewed retrospectively by three radiologists with experience in abdominal radiology in consensus reading. One radiologist had 10 years of experience (S.H.P) and the remaining two radiologists (S.J.Y., H.J.L.) had 3 years of experience at the time of the study. The data were classified according to specific organs, examination types, resident’s grade (i.e., years of training), presence of important findings, and discrepancies with and without legal consequences. Important findings of the abdomen were defined as the presence of a potentially life-threatening condition that may require immediate clinical management [9, 10]: 1) presence of active bleeding, 2) bowel obstruction, 3) organ ischemia or infarction, or 4) organ rupture based on the modification of critical results in abdominal radiology [11–17]. The definitions and descriptions of findings 1–4 are summarized in S1 Table.

The preliminary reports were evaluated for discrepancies of the final interpretations, including final reports and clinical outcome (surgery with pathology, intervention, endoscopy, and medical treatment based on EMR). The final reports were completed within 1–2 days after the preliminary report by one of four abdominal attending radiologists. The clinical outcome was reviewed on electronic medical records and classified as surgery, interventional treatment, endoscopic procedure, or medical treatment with clinical follow-up. The final reports with clinical outcome (i.e., final interpretations) were used as the reference standard for resident on-call reading.

### Imaging protocol

CT examinations were performed using a 64-section CT scanner (SOMATOM Definition, SOMATOM Definition Edge, Siemens Healthineers) and 128-section dual source CT scanners (SOMATOM Definition Flash, Siemens Healthineers). Four types of images were obtained: precontrast CT only, portal venous phase only (postcontrast), arterial and portal venous phase (postcontrast), precontrast, arterial, and portal venous phase (precontrast and postcontrast scans).

MRI examinations were performed for evaluation of acute appendicitis in pregnant patients and magnetic resonance cholangiopancreatography using a 3T scanner (Skyra, Siemens Healthineers).

Appendix US and upper abdomen US were included in the US examination.

### Statistical analysis

Residents were grouped as “discrepant” when the preliminary report differed from the final interpretation and as “identical” when the reports agreed. Patient characteristics in each group were compared using Student’s *t*-test and chi-square test. Univariable and multivariable logistic regression analyses were used to evaluate the risk factors of discrepancy interpretations by on-call residents, adjusting for covariates. Parameters with a *p* value less than 0.2 on univariable analysis, were included in the multivariable analysis [18, 19]. Multivariate logistic regression analysis was performed using the backward likelihood ratio. Differences were considered statistically significant with a 95% confidence interval and *p* < 0.050. All statistical analyses were performed using the SPSS software (version 22.0, IBM).

## Results

### Patient and interpretation characteristics

The clinical characteristics of the patients included in our study are summarized in Table 1. Of the 1132 patients, 544 (48.1%) were men and 588 (51.9%) were women, with a mean age ± standard deviation of 58.9 years ± 19.4 year. CT was the most common examination (*n* = 1064, 94.0%), followed by US (*n* = 49, 4.3%), MRI (*n* = 11, 1.0%), X-ray (*n* = 4, 0.4%), CT and MRI (*n* = 2, 0.2%), and MRI and US (*n* = 2, 0.2%). Among them, 678 patients (59.9%) underwent initial examinations, and 454 (40.1%) underwent follow-up examinations. Of the patients, 381 (33.7%) were inpatients and 751 (66.3%) were admitted to the emergency department. Among the 751 emergencies, 24 patients presented several lesions after traumatic accidents. Sixty-two patients were evaluated using 62 US or MRI, of which 50 (80.6%) were evaluated for acute appendicitis.

**Table 1:** Characteristics of patients with examinations interpreted by on-call residents.

Variables	Total	Identical	Discrepancy	*p-*value
	(N = 1132)	(N = 1068)	(N = 64)	
Age (years)	58.9 ± 19.4	58.8 ± 19.5	60.2 ± 22.0	0.605
Men: women	544: 588	516: 552	28: 36	0.244
Examinations				0.782
CT	1064 (94.0)	1002 (93.8)	62 (96.9)	
US	49 (4.3)	47 (4.4)	2 (3.1)	
MRI	11 (1.0)	11 (1.0)	0 (0)	
X-ray	4 (0.4)	4 (0.4)	0 (0)	
CT and MRI	2 (0.2)	0 (0.2)	0 (0)	
MRI and US	2 (0.2)	2 (0)	0 (0)	
CT Subgroups				0.063
Precontrast CT	36 (3.4)	34 (3.4)	2 (3.2)	
Postcontrast CT	405 (38.1)	390 (38.9)	15 (24.2)	
Pre and postcontrast CT*	623 (58.6)	578 (57.7)	45 (72.6)	
Initial examination	678 (59.9)	637 (59.6)	41 (64.1)	0.514
Patient class				0.447
Inpatient	381 (33.7)	356 (33.3)	25 (39.1)	
ED patient	751 (66.3)	712 (66.7)	39 (60.9)	
Year of residency				0.017
1	187 (16.5)	177 (16.6)	10 (15.6)	
2	432 (38.2)	396 (37.1)	36 (56.2)	
3	443 (39.1)	428 (40.1)	15 (23.4)	
4	70 (6.2)	67 (6.3)	3 (4.7)	
Organ				0.565
Bowel	567 (50.1)	530 (49.6)	37 (57.8)	
GB/BD/pancreas	139 (12.3)	131 (12.3)	9 (14.1)	
Liver	116 (10.2)	109 (10.2)	5 (7.8)	
KUB	99 (8.7)	97 (9.1)	2 (3.1)	
Peritoneum	47 (4.2)	46 (4.3)	1 (1.6)	
Ovary/uterus	45 (4.0)	41 (3.8)	4 (6.2)	
Retroperitoneum	31 (2.7)	30 (2.8)	1 (1.6)	
Muscle/wall/skin	30 (2.7)	29 (2.7)	1 (1.6)	
Vessel	23 (2.0)	23 (2.2)	0 (0)	
Spleen	9 (0.8)	8 (0.8)	2 (3.1)	
Lung	9 (0.8)	9 (0.8)	0 (0)	
Etc.	17 (1.5)	15 (1.4)	2 (3.1)	
Important finding	359 (31.7)	329 (30.8)	30 (46.9)	0.007
Final interpretations ^†^				0.106
Report, surgery/pathology	202 (17.8)	184 (17.2)	18 (28.1)	
Report, intervention	244 (21.6)	229 (21.4)	15 (23.4)	
Report, endoscopy	47 (4.2)	44 (4.1)	3 (4.7)	
Report, medical treatment	639 (56.4)	611 (57.2)	28 (43.8)	

Values are presented as number (%).

*Pre-and postcontrast CT mean precontrast and postcontrast CT.

^†^Report refers to the attending radiologist’s report.

ED, emergency department; CT, computed tomography; US, ultrasonography; MRI, magnetic resonance imaging; GB, gallbladder; BD, bile duct; KUB, kidney-ureter-bladder.

Fourteen residents, from 1^st^ year to 4^th^ year, had on-call duties during the study period. Preliminary reports from third year residents were the most common (*n* = 443, 39.1%) reports, followed by 2^nd^ year (*n* = 432, 38.2%), 1^st^ year (*n* = 187, 16.5%), and 4^th^ year (*n* = 70, 6.2%) residents. A total of 77 out of 187 cases were interpreted by 1^st^ year residents with assistance from senior residents. The bowel (n = 567, 50.1%) was the most common organ evaluated by on-call residents, followed by gallbladder/bile duct/pancreas (n = 139, 12.3%), and liver (n = 116, 10.2%). Final interpretations were determined using the attending radiologist reports surgery (n = 202, 17.8%), interventional treatment (n = 244, 21.6%), endoscopy (n = 47, 4.2%), or medical treatment (n = 639, 56.4%).

### Characteristics of discrepant interpretation

**Fig 2:**
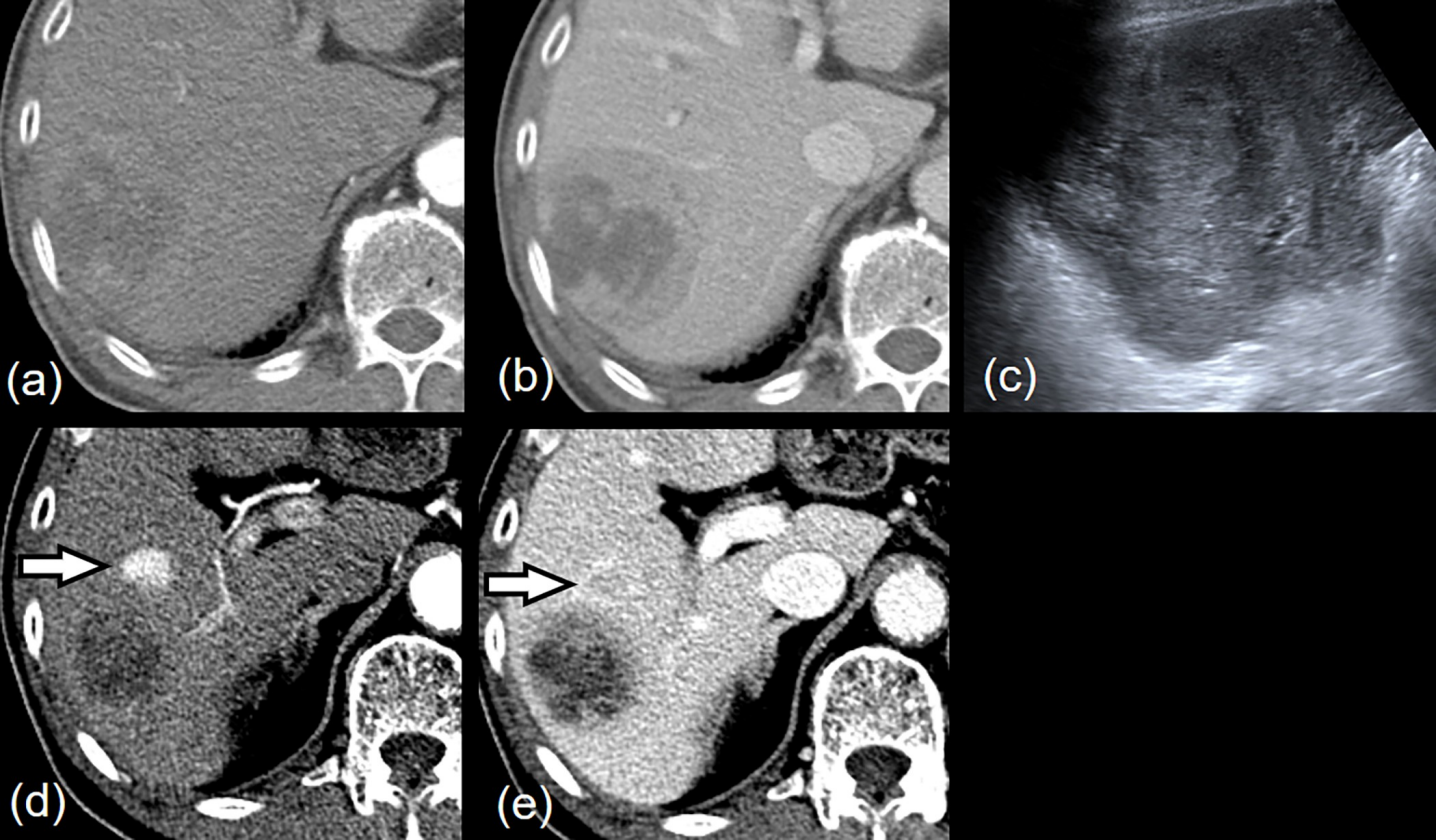
Discrepancy in a case between preliminary and final interpretations confirmed hepatocellular carcinoma. A 79-year-old man showed fever and right flank pain. **(A, B)** CT images show thick rim enhancing mass in the right posterior section of the liver. The heterogeneously enhancing mass in the liver was mistaken for liver abscess by the on-call resident. **(C)** The lesion shows a heterogeneous echoic mass in abdominal ultrasonography. **(D)** Another small arterial enhancing nodule in the inferior aspect of the lesion with washout **(E)** in the portal venous phase. Attending radiologist reported HCC in the right posterior section with additional HCC. A subsequent biopsy revealed HCC.

We found a total of 64 discrepancies (5.6%) between the preliminary reports from the residents and the final interpretations: acute appendicitis (*n* = 4), acute cholecystitis (*n* = 4), absence of abnormal bowel wall thickening (*n* = 4), colitis (*n* = 3), hepatocellular carcinoma (*n* = 3), normal appendix (*n* = 2), Crohn’s disease (*n* = 2), bile duct stone (*n* = 2), pancreatic cancer (*n* = 1), large gastric ulcer (*n* = 1), acute diverticulitis (*n* = 1), hemobilia (*n* = 1), ovarian torsion (*n* = 1), ovarian abscess (*n* = 1), hemorrhagic ovarian cyst (*n* = 1), urothelial cancer (*n* = 1), fungal infection in the spleen (*n* = 1), prostatic abscess (*n* = 1), and 30 important discrepant cases on final interpretations. Of these 64, the majority of imaging modalities were CT (*n* = 62, 96.9%) except for two USs in pregnant patients with acute appendicitis. The bowel (*n* = 37, 57.8%) was the most frequently discrepant organ, followed by gallbladder/bile duct/pancreas (*n* = 9, 14.1%), liver (*n* = 5, 7.8%, Fig 2), and others. Of the 64, 33 patients underwent delayed surgery (*n* = 18, 28.1%) or interventional treatment (*n* = 15, 23.4%). The preliminary report by 2^nd^ year resident showed the highest number of discrepancies (*n* = 36, 56.2%) compared the other residency years (*p* = 0.017).

### Characteristics of preliminary reports with important findings

Tables 2 and 3 summarize the important findings reported by on-call residents. A total of 359 patients (31.7%) had disease with 379 important findings: active bleeding (*n* = 222), organ rupture (*n* = 77), bowel obstruction (*n* = 52), bowel ischemia (*n* = 24), and organ infarction (*n* = 4). Eighteen patients had two important findings, and one had three important findings.

**Table 2:** Characteristics of important finding interpretations by on-call residents.

Variables	Total	Identical	Discrepancy	*p-*value
	(N = 359)	(N = 329)	(N = 30)	
CT Examinations	357 (99.4)	327 (99.4)	30 (100)	0.668
Initial examination	191 (53.2)	175 (53.2)	16 (53.3)	0.988
Patient class				0.254
Inpatient	155 (43.2)	139 (42.2)	16 (53.3)	
ED patient	204 (56.8)	190 (57.8)	14 (46.7)	
Grade of residents				0.178
1	63 (19.1)	54 (16.4)	5 (16.7)	
2	130 (36.2)	114 (34.7)	16 (53.3)	
3	143 (39.8)	136 (41.3)	7 (23.3)	
4	27 (7.5)	25 (7.6)	2 (6.7)	
Important findings^†^ *				
Active bleeding	222 (61.8)	208 (63.2)	14 (46.7)	0.080
Bowel obstruction	52 (14.5)	42 (12.8)	10 (33.3)	0.002
Rupture of organ	77 (21.4)	71 (21.6)	6 (20.0)	0.840
Organ infarction	4 (1.1)	4 (1.2)	0	0.544
Bowel ischemia	24 (6.7)	22 (6.7)	2 (6.7)	0.997
Organ				0.999
Bowel	235 (65.5)	215 (65.3)	20 (66.7)	
Liver	25 (7.0)	23 (7.0)	2 (6.7)	
KUB	18 (5.0)	17 (5.2)	1 (3.3)	
Peritoneum	16 (4.5)	15 (4.6)	1 (3.3)	
Ovary/uterus	14 (3.9)	13 (4.0)	1 (3.3)	
Retroperitoneum	13 (3.6)	12 (3.6)	1 (3.3)	
GB/BD/pancreas	7 (1.9)	6 (1.8)	1 (3.3)	
Spleen	7 (1.9)	6 (1.8)	1 (3.3)	
Etc.	24 (6.7)	22 (6.7)	2 (7.7)	
Treatment				0.586
Surgery	81 (22.6)	72 (21.9)	11 (36.7)	
Intervention	99 (27.6)	90 (27.4)	9 (30.0)	
Endoscopy	28 (7.8)	27 (8.2)	1 (3.3)	
Medical treatment	151 (42.1)	140 (42.6)	9 (30.0)	

Note. ED, emergency department; Values are presented as number (%).

KUB, kidney-ureter-bladder; GB, gall bladder; BD, bile duct.

^†^ Of 359, eighteen patients had two important findings, and one had three important findings.

*Of 30, two patients had two important findings.

**Table 3:** Detailed characteristics of important findings by on-call residents.

	Active bleeding	Organ rupture	Bowel obstruction	Bowel ischemia	^†^Bowel obstruction and ischemia	^†^Active bleeding and organ rupture	Organ Infarction	^†^Bowel obstruction and rupture	*Bowel obstruction, ischemia, and active bleeding	Total
Bowel	118 (55.1)	51 (73.9)	40 (100)	13 (100)	10 (100)	1 (14.3)	0	1 (100)	1 (100)	235 (65.5)
Liver	15 (7.0)	4 (5.8)	0	0	0	5 (71.4)	1 (25.0)	0	0	25 (7.0)
KUB	9 (4.2)	5 (7.2)	0	0	0	1 (14.3)	3 (75.0)	0	0	18 (5.0)
Peritoneum	13 (6.1)	3 (4.3)	0	0	0	0	0	0	0	16 (4.5)
Ovary-uterus	11 (5.1)	3 (4.3)	0	0	0	0	0	0	0	14 (3.9)
Retroperitoneum	12 (5.6)	1 (1.4)	0	0	0	0	0	0	0	13 (3.6)
Muscle-wall	13 (6.1)	0	0	0	0	0	0	0	0	13 (3.6)
GB/BD/pancreas	5 (2.3)	2 (2.9)	0	0	0	0	0	0	0	7 (1.9)
Spleen	7 (3.3)	0	0	0	0	0	0	0	0	7 (1.9)
Vessel	3 (1.4)	0	0	0	0	0	0	0	0	3 (0.8)
Lung	1 (0.5)	0	0	0	0	0	0	0	0	1 (0.3)
Etc.	7 (3.3)	0	0	0	0	0	0	0	0	7 (1.9)
Total patient No.	214	69	40	13	10	7	4	1	1	359

Note. GB, gall bladder; BD, bile duct; KUB, kidney-ureter-bladder.

^†^Two important findings

*Three important findings.

Only thirty patients (2.6%) showed 32 important discrepant findings, including 14 active bleeding, ten bowel obstructions, six organ ruptures, and two bowel ischemia. Two patients had two important findings. Of 30, all cases were CT, initial CT examinations were 16 (53.3%), and bowel was the most common organ (20, 66.7%). Although 14 patients showed active bleeding on CT scans, the on-call residents were unable to detect it. In addition, 10 cases of bowel obstruction were mistaken as paralytic ileus (6 cases), pelvic inflammatory disease (1 case), absence of bowel perforation (1 case), acute diverticulitis (1 case), and paraduodenal hernia (1 case) in the preliminary reports. In the patient with paraduodenal hernia, the resident detected the transitional zone of the small bowel, but different diagnosis was interpreted during on-call duty. Therefore, of the 30 discrepant cases, 23 (76.7%) were perceptual errors (i.e., no detection of the transitional zone at bowel obstruction or active bleeding focus in preliminary readings) leading to misinterpretations. Perceptual errors were most frequently noted in preliminary reports with important discrepant findings. Table 4 provides details about the important discrepant cases. The patients’ management included surgery (11/30, 36.7%), interventional treatment (9/30, 30.0%), endoscopy (1/30, 3.3%), or medical treatment (9/30, 30.0%). However, there were no legal consequences related to the interpretation discrepancies.

**Table 4:** Detailed characteristics of important discrepant cases by on-call residents.

Pt No.	R	Y	Sex	Exam	Preliminary reports	Final interpretations	Treatment	Organ	Pt class	CT
1	4	78	M	CT	Paralytic ileus	Ischemic colitis at descending colon	Medical	Bowel	ED	Post
2	3	48	M	CT	No active bleeding, hemoperitoneum	Active bleeding and rupture of HCC	Intervention	Liver	In	Pre+post
3	2	37	F	CT	PID	Small bowel obstruction with ischemia	Surgery	Bowel	In	Post
4	2	62	M	CT	No active bleeding, hemoperitoneum	Active bleeding, pseudoaneurysm	Intervention	Spleen	ED	Post
5	3	67	F	CT	Paraduodenal hernia	Small bowel obstruction due to adhesive ileus	Medical	Bowel	In	Post
6	3	75	F	CT	Bowel obstruction	Small bowel perforation	Surgery	Bowel	ED	Pre+post
7	2	81	F	CT	Paralytic ileus	Small bowel obstruction due to bezoar	Surgery	Bowel	In	Post
8	2	88	M	CT	No bowel perforation	Gastric outlet obstruction with advanced gastric cancer	Intervention	Bowel	ED	Pre+post
9	2	47	F	CT	Paralytic ileus	Sigmoid colon cancer with perforation	Surgery	Bowel	ED	Post
10	1	44	F	CT	Acute diverticulitis	Small bowel obstruction due to adhesive ileus	Surgery	Bowel	ED	Pre+post
11	2	40	M	CT	Paralytic ileus	Small bowel obstruction due to adhesive ileus	Medical	Bowel	ED	Pre+post
12	2	93	F	CT	No active bleeding	Active bleeding, thigh muscle	Medical	Muscle	In	Pre+post
13	2	72	F	CT	No active bleeding	Active bleeding, around LT site	Intervention	Diaphragm	In	Pre+post
14	4	73	M	CT	Pneumatosis intestinalis	Rupture of pneumatosis intestinalis	Surgery	Bowel	ED	Pre+post
15	3	26	F	CT	No active bleeding	Active bleeding	Medical	Ovary-uterus	In	Pre+post
16	1	60	F	CT	No active bleeding	Active bleeding, stomach	Intervention	Bowel	In	Pre+post
17	3	79	M	CT	No active bleeding	Active bleeding, hemobilia	Medical	GB/BD/pancreas	In	Pre+post
18	2	82	M	CT	No active bleeding, prominent vessel in rectum	Active bleeding, rectum	Endoscopy	Bowel	In	Pre+post
19	3	34	F	CT	Paralytic ileus	Small bowel obstruction due to adhesive ileus	Medical	Bowel	ED	Pre+post
20	2	83	F	CT	Fecal impaction colon with paralytic ileus	Sigmoid colon cancer with bowel obstruction	Medical	Bowel	ED	Pre+post
21	2	55	M	CT	No active bleeding	Active bleeding, colon diverticulum	Intervention	Bowel	In	Pre+post
22	2	54	M	CT	Paralytic ileus	Small bowel obstruction due to omental seeding invasion	Medical	Bowel	ED	Post
23	2	63	M	CT	Sigmoid colon perforation, pneumoperitoneum	Advanced gastric cancer with perforation	Surgery	Bowel	ED	Pre+post
24	2	53	M	CT	No active bleeding	Active bleeding, pelvic cavity	Medical	Retroperitoneum	In	Post
25	3	75	M	CT	Paralytic ileus	Small bowel obstruction due to adhesive ileus	Surgery	Bowel	ED	Pre+post
26	1	62	M	CT	No active bleeding	Active bleeding, ileum	Intervention	Bowel	In	Pre+post
27	2	49	F	CT	No active bleeding, hematoma	Active bleeding, LT site	Intervention	Liver	In	Pre+post
28	1	67	F	CT	No active bleeding, hematoma	Active bleeding, kidney	Medical	KUB	In	Pre+post
29	1	56	M	CT	No active bleeding	Active bleeding, omentum	Surgery	Peritoneum	In	Pre+post
30	2	52	M	CT	Peritonitis, invisible bowel perforation	Sigmoid colon perforation due to abscess	Intervention	Bowel	ED	Pre+post

Pt, patient; R, Grade of residents; Y, year-old; Exam, examination; M, male; F, female; CT, computed tomography; PID, pelvic inflammatory disease; LT, liver transplantation; HCC, hepatocellular carcinoma; ED, emergency department; GB, gallbladder; BD, bile duct; KUB, kidney-ureter-bladder; In, inpatient; Post, Postcontrast CT; Pre+post, Pre and post CT.

Final interpretation was defined as final report with patient data including surgery (operation note, pathology), intervention, and medical treatment on EMR data.

#### Risk factors for predicting discrepancy interpretations by on-call residents

**Fig 3:**
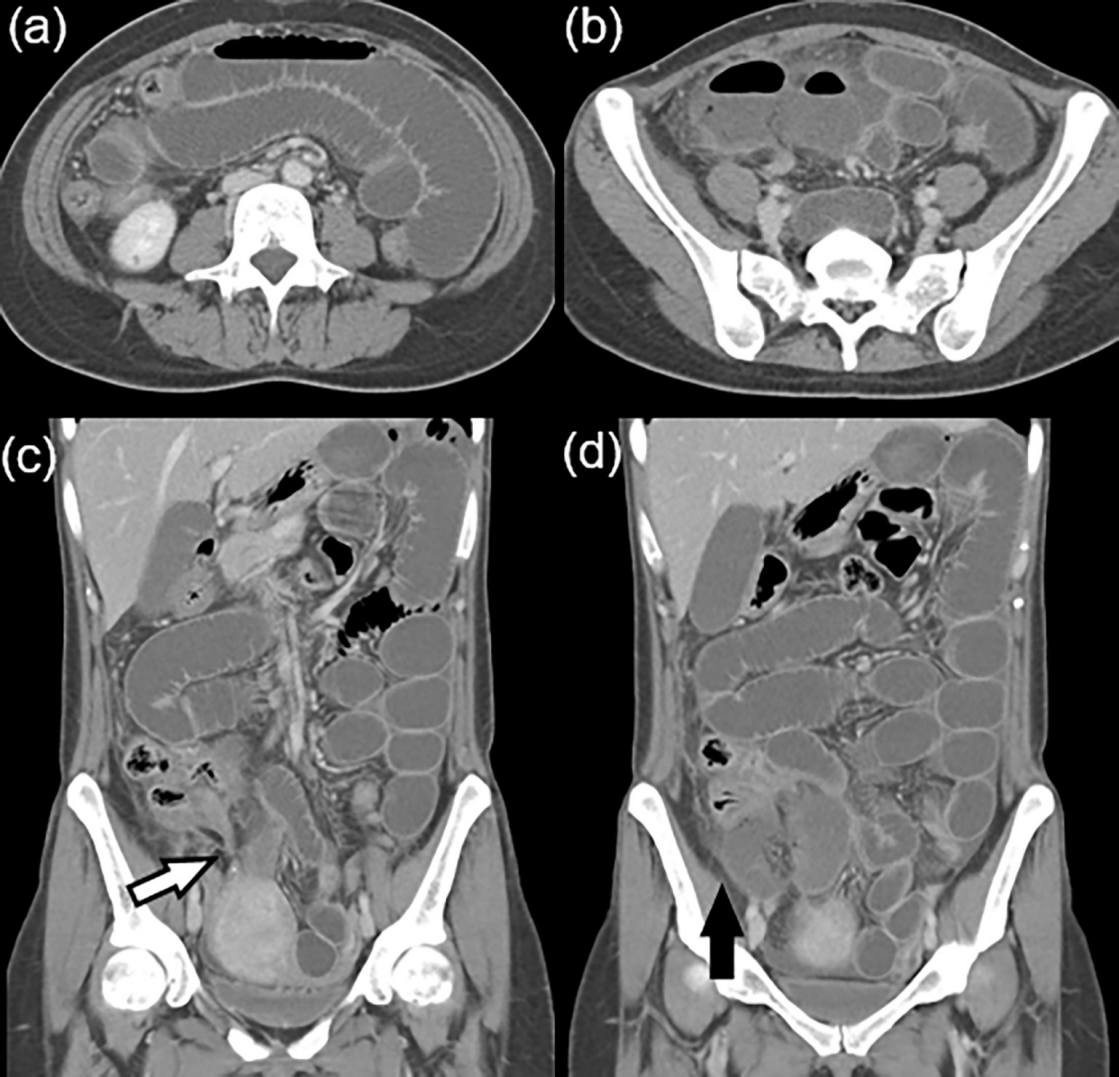
Important discrepant finding between preliminary and final interpretations confirmed small bowel obstruction with ischemia. A 39-year-old woman showed abdominal pain in the right lower quadrant area. The patient underwent an appendectomy 20 years ago and was treated for pelvic inflammatory disease a few years ago. The patient underwent abdominopelvic computed tomography (CT, single portal venous phase) in the emergency department. **(A, B)** Axial CT images show segmental distension of the small bowel. **(C)** In the coronal image, abrupt narrowing of dilated ileum is suggested to be the transitional zone (white arrow) of small bowel obstruction. **(D)** CT shows decrease in the segmental wall enhancement of small bowel, suggesting ischemic change (black arrow). The preliminary report by second-year radiology resident on duty indicated pelvic inflammatory disease with paralytic ileus. The patient continuously complained of abdominal pain with fever and underwent operation (operation finding: strangulated bowel obstruction), as resection of strangulated ileum. Histopathology revealed transmural necrosis of the ileum.

Table 5 shows risk factors for discrepancy between preliminary and final interpretations in the important findings (n = 359). The results of the univariable analysis showed a specific grade of residents (resident 2^nd^ year), and bowel obstruction (*p* < 0.2) were available risk factors included in the multivariable analysis. In the multivariable analysis, bowel obstruction (adjusted OR, 2.52; 95% CI: 1.00–6.50, *p* = 0.049) was an independent risk factor for important discrepant findings (Fig 3). Of 52 bowel obstruction interpretations, eight were interpreted by 1^st^ year (three discrepancies), 18 were interpreted by 2^nd^ year (four discrepancies), 21 were interpreted by 3^rd^ year (three discrepancies), and five were interpreted by 4^th^ year residents (no discrepancies).

**Table 5:** Risk factors for discrepancy in the important finding interpretations by on-call residents.

Variables	Univariable analysis		Multivariable analysis	
	Odd ratio (OR)	*P* Value	Adjusted OR	*p* Value
Important discrepancy				
Active bleeding	1.22 (0.24–6.28)	0.810		
Bowel obstruction	2.65 (1.01–6.97)	0.049	2.52 (1.00–6.50)	0.049
Rupture of organ	1.10 (0.38–3.13)	0.863		
Bowel ischemia	0.806 (0.15–4.49)	0.806		
Organ				
Liver	1.33 (0.28–6.42)	0.722		
Year of residents				
1, 3, 4	1			
2	1.94 (1.36–3.79)	0.110		

Note. Data in parentheses are 95% confidence intervals.

## Discussion

This study investigated the rate and risk factors of discrepancies between on-call residents’ and final interpretations considering the attending radiologist’s report and clinical outcomes on abdominal examinations. The rate of overall and important discrepancies was low in abdominal radiology. Bowel obstruction was a significant risk factor for important discrepant findings. The bowel showed the highest discrepancy. Educating abdominal residents emphasizing the bowel and bowel obstruction may improve the interpretation ability of radiology reports during on-call duty.

We found a 5.6% (64/1132) discrepancy rate between preliminary and final interpretations. Previous studies have reported an overall discrepancy rate from 0.1% to 3.8% [1–7, 20–25] in the preliminary radiology reports. Few studies [1, 3, 20] have reported that body CT may be associated with discrepant interpretations given the slightly higher discrepancy rate (6.4%, 9.8%, respectively) compared with the overall discrepancy rate [1, 3]. Our study analyzed abdominal cases, mainly abdominopelvic CT. Our rate (5.6%) was similar or slightly lower than that reported in previous studies. We consider that the resulting rates may reflect different clinical practice environments or educational efforts regarding the review of discrepant cases.

The bowel was the most common preliminary interpreted organ and had the highest discrepancy rate in our study. The high frequency of bowel interpretations during on-call duty may be explained by the common pathologies of acute abdominal pain, including gastrointestinal perforation or inflammation and bowel obstruction or infarction [26]. This finding was similar to that in a previous study regarding abdominal and pelvic CT taken in an emergency department, where bowel disease showed the highest discrepancy between preliminary and final reports [25]. Another study suggested that acute appendicitis in contrast-enhanced abdominopelvic CT was the most common cause of misinterpretation [20]. Considering previous studies and our present study, residents should pay urgent attention to the evaluation of bowel disease during on-call duty and study radiologic findings of this pathology before starting and during their after-hour coverage.

We found that bowel obstruction was significantly associated with discrepant preliminary and final interpretations, with an adjusted OR of 2.52. Abdominal CT is an important diagnostic modality for detecting small bowel obstruction and predicting surgical candidates [27, 28]. The CT findings of small bowel obstruction were feces signs, transitional zones, beak signs, mesenteric vessel course, presence of closed-loop obstruction or ischemia, and ascites [12, 28, 29]. The radiologist can detect the transitional zone between the dilated and collapsed loops using a bowel trace on consecutive CT images. One possible explanation for our results is that the bowel tracing skills to find the transitional zone (i.e., obstruction site) are acquired through a relatively long learning curve, which may have affected the preliminary report results. Our results also showed misinterpretations by 1^st^ and 2^nd^ year residents were higher than those by 3^rd^ and 4^th^ year residents. Although next-day CT readings by the abdominal attending radiologist can minimize the patient severity risk, performing early accurate diagnosis of bowel obstruction on the preliminary report may improve the patient care because delayed surgical management of bowel obstruction can increase the mortality and morbidity rates of patients and prolong hospitalization [30]. Additional practice before and during on-call duty is thus essential to identify the number and location as well as the presence of transitional zones related to closed-loop small bowel obstruction and development of pneumoperitoneum, pneumatosis intestinalis, and portal vein gas, which is highly suspected to be a surgical candidate and complications of bowel obstruction [28].

Among the 1132 evaluated cases, our results showed 30 important discrepant findings categorized into active bleeding, bowel obstruction, organ ischemia or infarction, and organ rupture. We found that perceptual errors during preliminary interpretation were the most common cause of important discrepancies. Perceptual errors develop during initial screening (i.e., failure to recognize an abnormality) and cause missed diagnoses in radiology. Consistent with our results, perception errors have been reported to be the most common and important mistake made by radiologists [8, 31, 32]. We suggest residents to collect and review missed lesions showing important findings on CT to reduce the error incidence and improve the diagnostic accuracy. We believe that education can improve radiologic interpretations throughout training. Critical point of the important findings obtained with imaging modalities may require surgical or interventional approaches. As important discrepant findings are directly related to life-threatening scenarios, our educational goal should be aimed at reducing the frequency of discrepancies.

Our study further showed that 446 patients (39.4%) underwent surgery or intervention. Among them, 33 patients (2.9%) underwent delayed surgery or interventional treatment after a preliminary radiology report. These results suggest that the discrepancy in on-call residents’ preliminary interpretations can lead to management changes. Similarly, previous studies have demonstrated that discrepancies in on-call residents’ preliminary interpretations can affect patient care and management [3, 20–24]. McWilliams et al. [22] studied abdominal imaging and other body-part imaging, finding that 44.6% of the discrepant preliminary cases resulted in management changes, and 14% of the discrepant preliminary cases caused therapeutic management changes, such as surgery and interventional endoscopic procedures, while 11.9% of the discharged patients were recalled. Ruchman et al. [20] suggested that 7.2% of discrepant reports showed a negative effect on patients. Friedman et al. [24] reported that 35.7% of such cases increased the patients’ morbidity and hospitalization period, whereas discrepant preliminary reports did not increase mortality or long-term outcomes.

We also found that the experience of a second-year resident was a possible risk factor for the discrepancies. However, a specific training degree was not a significant risk factor after multivariable analysis. In our study, it was difficult to evaluate the experience of a first-year resident because the number of overnight duty days in the first-year was small and they can ask a senior resident regarding the difficult case. These results may differ from a previous study [20], which showed the highest discrepancy rate for residents who were in their third year of training. Mellnick et al. [5] reported that a higher grade of residents led to more discrepancies, whereas other studies reported that a higher grade of residents led to reduced discrepancies [1, 3, 6, 7]. We suggest that the overnight coverage ratio in a specific residency year and different education systems depending on the academic institutions can affect the discrepancy results. In addition, training programs have undergone many changes over the years, including strict work-hour regulations in South Korea, increased training under supervision, and decreased trainee independence [7, 33]. Few studies have reported higher error rates in residents working more than 10 consecutive hours overnight [34] and increasing their caseload or working hours [35], and these error rates may be associated with fatigue or circadian effects.

This study has various limitations. First, it was a retrospective single-center study in South Korean population that inevitably leads to selection bias. Second, our study was performed in a tertiary academic medical institution including regional emergency medical, cancer, and trauma centers for a specific region, possibly impacting the severity of cases in enrolled patients. Third, discrepancies were noted only for a small portion of patients. Thus, our study revealed one risk factor. Including more patients may be conducive to identify additional risk factors. Finally, only a small number of imaging modalities besides CT were considered in this study.

In conclusion, overall and important discrepant findings between preliminary interpretations by on-call residents and final interpretations showed a low rate in abdominal radiology. Nevertheless, bowel obstruction is a risk factor for discrepancies, and the bowel is the most common target of on-call interpretations.

## Supporting information

S1 Checklist STROBE statement. (DOC)

S1 Table Important findings and definitions. (DOCX)
