# Supplementary material for: Interpretation discrepancies of abdominal imaging by on-call radiology residents: Evaluation of risk factors
Source: PLoS One. 2022 Sep 9;17(9):e0274313. doi: 10.1371/journal.pone.0274313 (PMC9462765; doi:10.1371/journal.pone.0274313)
Supplement: S1 Table — (DOCX) [file pone.0274313.s002.docx]

Supplementary Table 1. Important findings and definitions

| Important findings | Definitions ^*^ |
| --- | --- |
| 1) Active bleeding | Active contrast extravasation and pseudoaneurysm [11] |
| 2) Bowel obstruction | Transition zone (proximal loop dilatation and collapse or normal caliber of distal loops) with or without obstructive lesions [12-13] |
| 3-1) Bowel ischemia | Decreased or absent bowel enhancement [14] |
| 3-2) Organ infarction | Multiple foci of non-enhancement areas in the visceral organ; typically wedge-shaped infarcted areas[15] |
| 4) Organ rupture | Indistinct margin of the subcapsular area of the organ with hematoma/hemoperitoneum or bowel perforation with pneumoperitoneum [16-17] |

^*^ The important findings and definitions were described on the abdominopelvic CT examinations.
